# Capillary Electrophoretic Enantioseparation of Profens: Advances in Chiral Selectors, Separation Strategies, and Analytical Applications

**DOI:** 10.3390/molecules31183256

**Published:** 2026-09-14

**Authors:** Gabriel Hancu, Maria Matean, Anca Gabriela Cârje

**Affiliations:** 1 Department of Pharmaceutical and Therapeutic Chemistry, Faculty of Pharmacy, “George Emil Palade” University of Medicine, Pharmacy, Science and Technology of Târgu Mures, 540120 Târgu Mures, Romania; 2 Department of Analytical Chemistry and Drug Analysis, Faculty of Pharmacy, “George Emil Palade” University of Medicine, Pharmacy, Science and Technology of Târgu Mures, 540120 Târgu Mures, Romania; maria_matean@yahoo.com (M.M.); anca.carje@umfst.ro (A.G.C.)

**Keywords:** capillary electrophoresis, chirality, chiral selectors, cyclodextrins, enantioseparation, NSAIDs, profens, enantiomeric purity

## Abstract

Profens are chiral nonsteroidal anti-inflammatory drugs (NSAIDs) whose enantiomers may differ in pharmacokinetics, pharmacological activity and pharmacotoxicological profile, making enantioselective analysis relevant for pharmaceutical quality control and bioanalytical applications. Capillary electrophoresis (CE) has been extensively investigated for their enantioseparation because of its high efficiency, low sample and reagent consumption, and the possibility of easily modifying the chiral recognition environment. This review traces the development of CE methods for profen enantioseparation from early exploratory studies focused on chiral selector (CS) screening to the most recent reports available up to 2026, highlighting the progressive shift toward quantitative, validated, and application-oriented methods. The influence of CS type and concentration, background electrolyte (BGE) composition and pH, organic modifiers, polarity, and capillary surface modification is discussed in relation to enantioselectivity and analytical performance. Although cyclodextrin (CD)-based systems remain predominant, the available literature shows that profen enantioseparation is strongly analyte-dependent and that no single CS or set of conditions is universally applicable. The review also examines the transition from proof-of-concept enantioseparation toward validated methods for enantiomeric impurity determination, configurational stability assessment, and the analysis of pharmaceutical, biological, and environmental samples, while highlighting the analytical performance and current limitations of CE-based approaches. Future developments should focus on improving sensitivity, robustness, and applicability to complex matrices while making greater use of rational CS selection, multivariate optimization, and complementary detection approaches.

## 1. Introduction

Chirality is an important feature of many pharmaceutical compounds, as chiral drugs represent a substantial proportion of marketed drugs. The enantiomers of a chiral drug may differ in their pharmacokinetic, pharmacodynamic and pharmacotoxicological properties because of stereoselective interactions with biological targets, including enzymes, receptors, transporters, and metabolic pathways. Such differences may influence therapeutic activity, drug disposition, and safety and underline the importance of considering stereochemistry during drug development, quality control, and clinical use [1,2].

Among chiral pharmaceuticals, nonsteroidal anti-inflammatory drugs (NSAIDs) represent an important therapeutic group in which stereochemistry influences pharmacological activity. A prominent subgroup comprises the 2-arylpropionic acid derivatives, commonly referred to as profens, which include widely prescribed drugs such as ibuprofen, ketoprofen, naproxen, flurbiprofen, or fenoprofen. Profens possess a stereogenic center at the α-carbon of the propionic acid moiety and therefore occur as two enantiomers, which may differ in their pharmacological activity and metabolic behavior [3,4].

**Figure 1:**
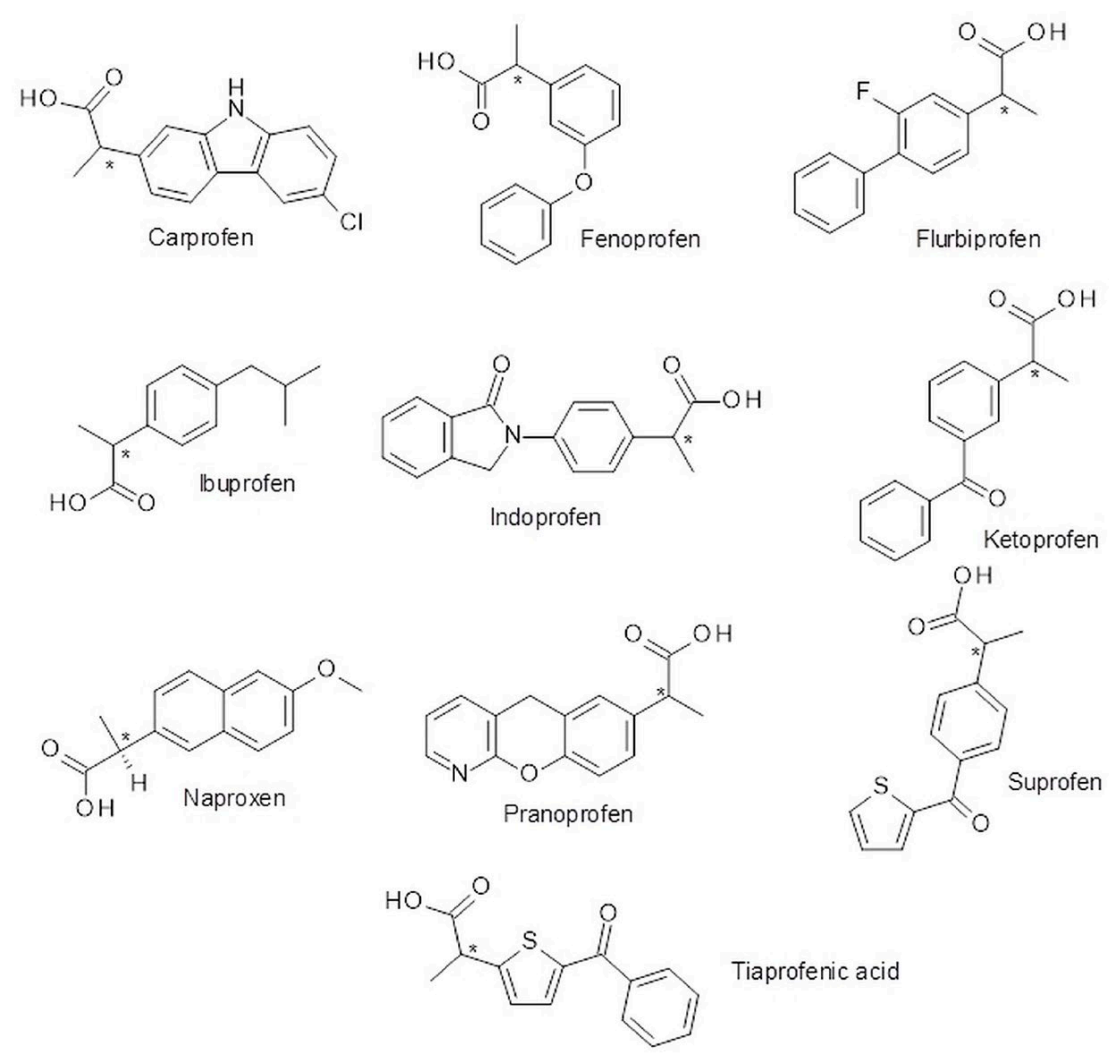
Chemical structures of the main 2-arylpropionic acid derivatives discussed in this review. The stereogenic centers are indicated by an asterisk (*).

The chemical structures of the main profens discussed in this review are presented in Figure 1.

**Figure 2:**
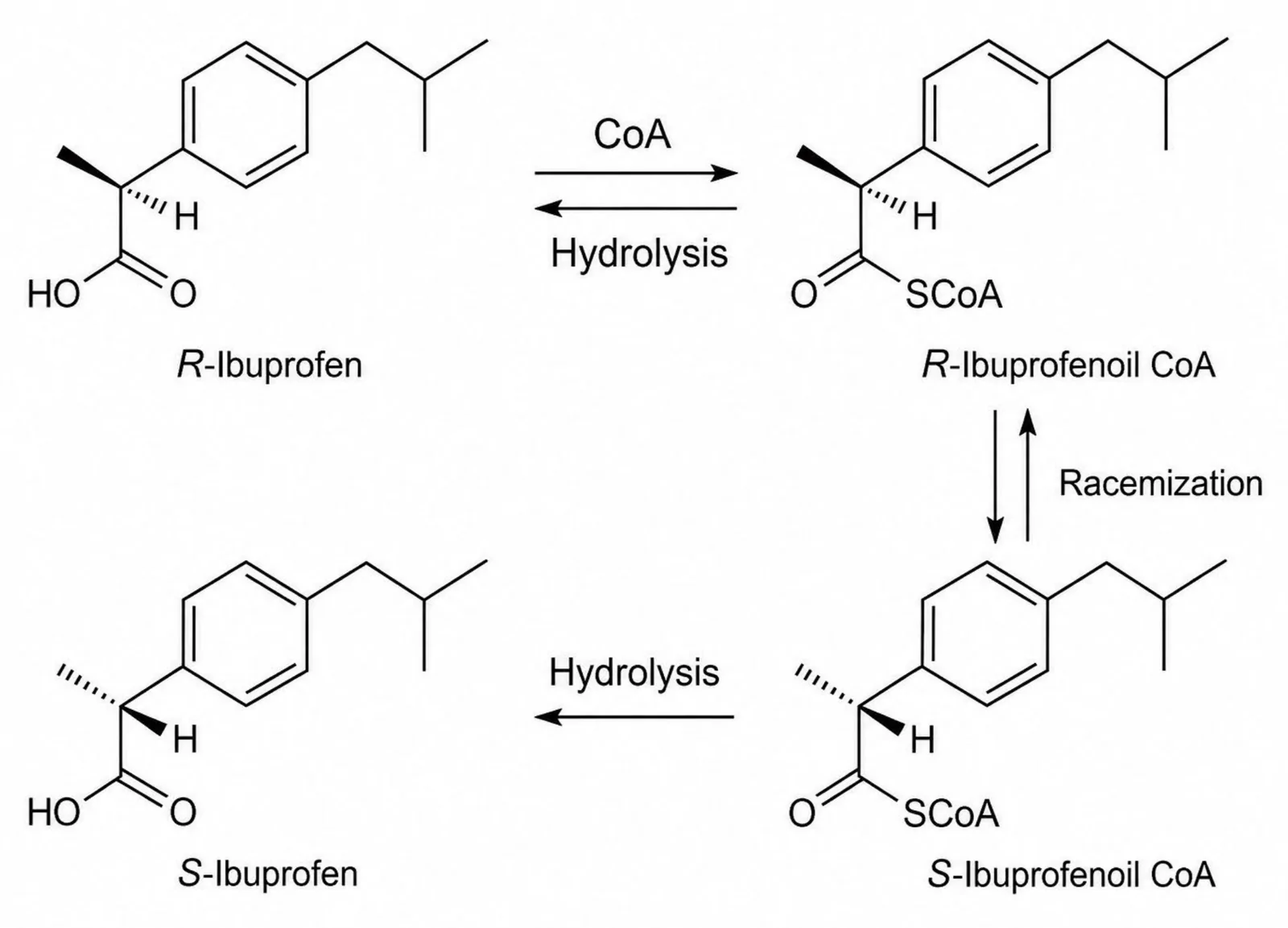
Metabolic chiral inversion of R-ibuprofen to S-ibuprofen via CoA thioester intermediates.

For most profens, inhibition of prostaglandin synthesis through cyclooxygenase (COX) inhibition is predominantly associated with the *S*-enantiomer, whereas the *R*-enantiomer generally shows lower activity toward this pathway [5]. However, the stereochemical behavior of profens is complicated by metabolic chiral inversion, whereby the *R*-enantiomer can be converted in vivo to the pharmacologically more active *S*-form. This process is generally unidirectional and is both compound- and species-dependent, with ibuprofen representing the best-characterized example, while other profens may undergo little or no inversion [6]. The unidirectional metabolic inversion of *R*-ibuprofen to *S*-ibuprofen is illustrated in Figure 2.

Consequently, the two enantiomers cannot be regarded simply as pharmacologically equivalent components of a racemate, and their individual determination is relevant to the assessment of pharmacological activity, pharmacokinetics, enantiomeric purity, and configurational stability.

From a pharmaceutical perspective, profens provide representative examples of the different approaches used for the development and clinical use of chiral drugs. Most members of this class were originally introduced as racemic mixtures, and several, including fenoprofen, flurbiprofen, and tiaprofenic acid, continue to be marketed as racemic drugs [7]. Naproxen represents a notable exception, being used as *S*-naproxen, which accounts for its anti-inflammatory activity, whereas *R*-naproxen exhibits substantially lower COX-inhibitory activity and has been associated with hepatotoxicity, making control of the enantiomeric impurity particularly important [8]. Ibuprofen and ketoprofen illustrate a third situation, as both were initially developed and marketed as racemic drugs and subsequently underwent a chiral switch. Their pharmacologically active *S*-enantiomers were later introduced as dexibuprofen and dexketoprofen, respectively, while the original racemic formulations have remained in clinical use [9]. Thus, within the profen class, racemic drugs, single-enantiomer drugs, and racemates accompanied by their chiral-switched single-enantiomer counterparts can all be encountered in therapeutic use [10].

The main chemical and stereochemical characteristics of the representative profens discussed in this review are summarized in Table 1.

These stereochemical particularities make enantioselective analysis particularly relevant for profens. The separate determination of individual enantiomers is required not only for the assessment of enantiomeric purity and the control of stereoisomeric impurities, but also for pharmacokinetic and metabolic studies, investigation of chiral inversion, and evaluation of the stereochemical stability of single-enantiomer formulations. Consequently, reliable chiral analytical methods are essential throughout drug development, quality control, and bioanalytical investigations [11,12].

Among the analytical techniques available for chiral analysis, capillary electrophoresis (CE) has emerged as a particularly versatile approach, offering high separation efficiency, low sample and reagent consumption, and considerable flexibility in chiral recognition through the direct addition of chiral selectors (CSs) to the background electrolyte (BGE) [13].

CE is particularly well suited to the enantioseparation of profens because their acidic character allows their electrophoretic behavior to be readily controlled by BGE pH, while a wide range of CSs can be screened and combined without the need for dedicated chiral stationary phases (CSPs). Cyclodextrins (CDs) and their derivatives have been the most extensively employed CSs for this purpose, although macrocyclic antibiotics, proteins, polysaccharides, ionic liquids (ILs), and more complex dual-selector systems have also been investigated [14,15].

In most chiral CE systems, enantioseparation results from differences in the apparent electrophoretic mobilities of the two enantiomers arising from stereoselective interactions with the CS and the transient formation of diastereomeric complexes. Differences in the binding affinities of the enantiomers toward the CS and/or in the electrophoretic mobilities of the resulting complexes provide the basis for enantiodiscrimination. Consequently, the nature and concentration of the CS, together with BGE pH and composition and other electrophoretic parameters, can be adjusted to optimize enantioselectivity and resolution [16].

Previous reviews have addressed the application of CE to profen and NSAID analysis from different perspectives. Patel et al. reviewed electrophoretic approaches for the enantioseparation of profen NSAIDs, providing an overview of the separation principles and experimental strategies used for this structurally related group of chiral drugs. Attention was given to CD-based systems, while alternative CSs and chiral capillary electrochromatography (CEC) approaches were also considered [17]. Macià et al. subsequently provided a broader overview of CE methods for NSAID analysis, encompassing both achiral and chiral applications and discussing their use in pharmaceutical, biological, and environmental samples [18]. Podar et al. focused more specifically on CE enantioseparation of NSAIDs, covering studies published between 2000 and 2015. Their review confirmed the predominant role of CDs and their derivatives as CSs, while also documenting the increasing diversification of chiral recognition strategies using alternative CSs and combined selector systems [19]. Together, these reviews established the analytical potential of CE for profen analysis and documented the progressive development of increasingly diverse approaches to chiral recognition.

However, a considerable period has elapsed since the literature covered by these reviews, during which CE-based profen enantioseparation has continued to develop in terms of both chiral recognition strategies and analytical applications.

The present review therefore critically examines the development of CE approaches for profen enantioseparation, from early studies to the most recent developments reported in 2026. While Patel et al. [17] primarily addressed electrophoretic enantioseparation approaches for profen NSAIDs, Macià et al. [18] provided a broader overview of both achiral and chiral CE applications for NSAIDs, and Podar et al. [19] reviewed CE enantioseparation of NSAIDs up to 2015, the present review integrates subsequent methodological developments with earlier work in a profen-specific framework. Emphasis is placed on the evolution from conventional single-CD systems toward dual-selector strategies, nonaqueous media, alternative CSs, modified capillaries, and stationary-phase-based approaches, as well as on the transition from proof-of-concept enantioseparation toward validated pharmaceutical, biological, and environmental applications.

## 2. Capillary Electrophoretic Approaches for Profen Enantioseparation

The following section provides a chronological overview of the development of CE methods for profen enantioseparation, covering the evolution from conventional CD-based systems to alternative CSs, dual-selector approaches, modified capillaries, and more complex chiral recognition systems.

An early alternative to CD-based chiral recognition was reported by D’Hulst & Verbeke, who investigated maltodextrins and related maltooligosaccharides as chiral electrolyte modifiers for the CE separation of profens. Maltodextrin mixtures provided effective enantiodiscrimination, particularly for ibuprofen, whereas flurbiprofen was more difficult to resolve and ketoprofen was not separated under the conditions investigated. Enantioselectivity was strongly influenced by the qualitative and quantitative composition of the maltodextrin mixtures, their concentration, and degree of hydrolysis [20].

Guttman & Cooke developed a quantitative chiral capillary gel electrophoresis (CGE) method for the separation and purity assessment of *R*- and *S*-naproxen. A replaceable non-cross-linked hydrophilic polymer network containing hydroxypropyl-β-cyclodextrin (HP-β-CD) as CS was used. Optimization of BGE pH, CS concentration, electric field strength, and temperature resulted in baseline enantioseparation within 15 min. The method allowed the detection of small variations in the distomer *R*-naproxen content of *S*-naproxen samples, supporting its application to enantiomeric purity control [21].

Fanali & Aturki investigated the enantioseparation of six profens (fenoprofen, flurbiprofen, ibuprofen, indoprofen, ketoprofen, and suprofen) by CZE using derivatized β-CDs as CSs. The effects of CS type and concentration, BGE pH, and MeOH content on enantiomeric resolution (Rs) were systematically evaluated. Among the tested CSs, heptakis-2,3,6-tri-O-methyl-β-cyclodextrin (Tri-OMe-β-CD) showed the highest stereoselectivity, providing baseline separation of all six profens under optimized conditions. The method was also applied to the qualitative analysis of commercial ibuprofen and ketoprofen formulations [22].

Lelièvre & Gareil investigated the enantioseparation of seven profens (carprofen, flurbiprofen, indoprofen, ketoprofen, naproxen, pranoprofen, and suprofen) by CZE using five neutral CDs as CSs. The effects of BGE pH and ionic strength, as well as CS type and concentration, on Rs were evaluated together with the determination of analyte pKa values and CD inclusion constants. Trimethyl-β-cyclodextrin (TM-β-CD) at pH 4.0 provided the best overall stereoselectivity, achieving baseline separation for most analytes. Interestingly, the stability of the CD inclusion complexes did not correlate directly with stereoselectivity, indicating that complex stability alone does not determine chiral recognition [23].

Fillet et al. investigated the enantioseparation of nine profens (carprofen, fenoprofen, flurbiprofen, ibuprofen, indoprofen, ketoprofen, sulindac, suprofen, and tiaprofenic acid) using dual-CD systems combining an anionic CD, sulfobutyl ether-β-cyclodextrin (SBE-β-CD) or carboxymethyl-β-cyclodextrin (CM-β-CD), with a neutral β-CD derivative (TM-β-CD). The anionic CD primarily acted as a carrier, imparting electrophoretic mobility to the analytes, while the neutral CD provided stereoselective recognition. The SBE-β-CD/TM-β-CD combination was the most effective, providing complete enantioseparation of all investigated NSAIDs within 15 min, with Rs values reaching 30.6 for carprofen [24].

Fanali et al. compared three CSs, Tri-OMe-β-CD, heptamethylamino-β-cyclodextrin (Hepta-MeNH-β-CD), and vancomycin, for the enantioseparation of eight profens (carprofen, cicloprofen, flurbiprofen, ibuprofen, indoprofen, ketoprofen, naproxen and suprofen) by CE. The effects of CS concentration and BGE pH on Rs were evaluated, together with limit of detection (LOD), linearity, repeatability, and separation efficiency. Vancomycin, used in the partial-filling mode, provided the shortest analysis time (<10 min) and the highest separation efficiency (up to 269,000 theoretical plates), while providing effective enantioseparation for most analytes [25].

Wang & Khaledi investigated quaternary ammonium β-cyclodextrin (QA-β-CD) as a cationic CS for nonaqueous capillary electrophoresis (NACE). The approach was evaluated for the enantioseparation of several profens (carprofen, fenoprofen, flurbiprofen, ibuprofen, indoprofen, ketoprofen, and suprofen), together with other chiral compounds. Formamide was the only solvent that enabled profen enantioseparation, whereas no separation was observed in water, MeOH, N-methylformamide, or dimethyl sulfoxide. The method was also applied to the qualitative analysis of a commercial ketoprofen formulation [26].

Blanco et al. developed a CZE method for the enantioseparation of fenoprofen, ibuprofen, and ketoprofen using CDs as CSs. The effects of CS type and concentration, BGE pH and concentration, temperature, and MeOH content on Rs were investigated. Tri-OMe-β-CD was the only tested CS that provided complete enantioseparation of all three profens. Under optimized conditions, baseline separation was achieved within 20 min, with Rs values of 4.76, 4.17, and 3.86 for fenoprofen, ibuprofen, and ketoprofen, respectively [27].

Wolbach et al. investigated quantitative structure–enantioselectivity relationships (QSER) for the CE separation of 22 profens, including fenoprofen, flurbiprofen, ibuprofen, ketoprofen, and suprofen. Three CSs, β-CD, HP-β-CD, and Tri-OMe-β-CD, were experimentally evaluated. Molecular descriptors related to size, shape, hydrophobicity, electronic properties, and hydrogen-bonding capacity were used in multivariate regression and neural-network models to predict the optimal CD, degree of enantioselectivity, and migration order. Tri-OMe-β-CD was the most effective CS for all five profens, showing the importance of analyte structure in determining CD-mediated enantiodiscrimination [28].

Abushoffa et al. investigated a dual-CD system combining the anionic heptakis-6-sulfato-β-cyclodextrin (HS-β-CD) with neutral Tri-OMe-β-CD for the enantioseparation of fenoprofen, flurbiprofen, ibuprofen, and ketoprofen. Affinity constants for each enantiomer toward both CDs were determined and used in mathematical models to predict enantioselectivity as a function of CS concentration. The dual-CD system provided complete enantioseparation of all four profens, with Rs values of 4.3, 2.9, 3.3, and 6.1 for fenoprofen, flurbiprofen, ibuprofen, and ketoprofen, respectively. The predicted selectivity values were in good agreement with the experimental results [29].

Cucinotta et al. investigated three hemispherodextrins as CSs for the CE enantioseparation of six profens (flurbiprofen, ibuprofen, indoprofen, ketoprofen, suprofen, and tiaprofenic acid). THALAH, THAMH, and THCMH were evaluated individually at low concentrations (0.30–3.0 mM) and as binary mixtures. Pronounced selector–analyte specificity was observed, with no single hemispherodextrin providing effective enantioseparation of all profens. The use of binary mixtures provided complementary stereoselectivity and improved the simultaneous separation of several racemates, particularly ibuprofen, suprofen, and tiaprofenic acid [30].

Simó et al. developed a quantitative chiral CE method for monitoring the release of *R*- and *S*-ibuprofen from polymeric drug-delivery systems. After evaluating β-CD- and maltodextrin-based systems, dextrin 10 was selected as CS in a sodium tetraborate BGE, providing enantioseparation within 5 min. The method was linear over 1–40 μg/mL, with a limit of quantification (LOQ) of 1 μg/mL for each enantiomer and allowed the detection of 0.5% *R*-ibuprofen in the presence of 99.5% *S*-ibuprofen. The method was subsequently applied to monitor ibuprofen release from two polymeric systems in phosphate buffer and rat plasma [31].

Blanco et al. developed and validated a CE method for the assessment of the enantiomeric purity of *S*-ketoprofen in oral pharmaceutical formulations using Tri-OMe-β-CD as CS. The method was designed for the determination of trace levels of *R*-ketoprofen, achieving an LOD of 0.04% relative to *S*-ketoprofen. Application to stability studies showed that the total ketoprofen content remained essentially unchanged during storage, while the *R*-ketoprofen impurity ranged from 0.1 to 0.4% [32].

Abushoffa et al. investigated a dual-CD system combining the cationic permethyl-6-monoamino-6-monodeoxy-β-CD (PMMA-β-CD) with the anionic HS-β-CD for the enantioseparation of fenoprofen, flurbiprofen, ibuprofen, and ketoprofen. The combination of oppositely charged CDs enhanced enantioselectivity, Rs, and separation efficiency compared with the individual CSs. Binding constants for each enantiomer–CD complex were determined and used to predict the optimal CS concentrations. Under optimized conditions, Rs values of 11.4, 6.5, 12.5, and 4.4 were obtained for fenoprofen, flurbiprofen, ibuprofen, and ketoprofen, respectively, with migration times of 12–21 min [33].

La et al. developed a chiral CE method for the simultaneous enantioseparation of nine profens (fenoprofen, flurbiprofen, ibuprofen, indoprofen, ketoprofen, naproxen, pirprofen, suprofen, and tiaprofenic acid) using two complementary approaches: a single Tri-OMe-β-CD system in normal polarity and a dual CM-β-CD/Tri-OMe-β-CD system in reversed polarity. The two modes produced opposite enantiomer migration orders, facilitating peak identification. The reversed polarity system generally provided higher Rs, reaching 8.0 for naproxen, compared with 4.4 in normal phase. The method also allowed the detection of 0.5% *R*-enantiomer in the presence of 99.5% of the corresponding *S*-enantiomer [34].

Dey et al. introduced the vesicle-forming cationic surfactant (1*R*,2*S*)-N-dodecyl-N-methylephedrinium bromide (DMEB) as CS for the MEKC enantioseparation of eight profens (carprofen, fenoprofen, flurbiprofen, ibuprofen, indoprofen, ketoprofen, naproxen, and suprofen). The effects of BGE pH and DMEB concentration on Rs were investigated. Enantioseparation was strongly influenced by electrostatic interactions between the anionic profens and the cationic vesicles, while hydrophobic interactions also contributed to analyte–vesicle association. Under optimized conditions, several profens and their enantiomers could be separated simultaneously in a single run [35].

Glowka & Karaźniewicz developed and validated a stereospecific CZE method for the simultaneous determination of flurbiprofen, ibuprofen, and ketoprofen enantiomers in human serum. Tri-OMe-β-CD was used as CS, providing baseline enantioseparation of all three profens in a single run following liquid–liquid extraction (LLE) from serum. The method was subsequently applied to pharmacokinetic and bioavailability studies of ketoprofen enantiomers after administration of conventional and sustained-release formulations [36].

Głowka & Karaźniewicz also developed a stereospecific CZE method for the quantitative determination of *R*- and *S*-ibuprofen in human serum and urine using Tri-OMe-β-CD as CS. Sample preparation was based on C18 solid-phase extraction (SPE), and complete enantioseparation was obtained with Rs values of 2.19 in serum and 2.25 in urine. The method was validated for both matrices and applied to urine samples collected after administration of 400 mg racemic ibuprofen to a healthy volunteer [37].

Fradi et al. investigated the NACE enantioseparation of several acidic pharmaceuticals, including fenoprofen, flurbiprofen, ibuprofen, indoprofen, ketoprofen, suprofen, and tiaprofenic acid, using single-isomer amino-β-CD derivatives as CSs. Three CSs were evaluated: 6-monodeoxy-6-mono(2-hydroxy)ethylamino-β-cyclodextrin (EA-β-CD), 6-monodeoxy-6-mono(2-hydroxy)propylamino-β-cyclodextrin (IPA-β-CD), and 6-monodeoxy-6-mono(3-hydroxy)propylamino-β-cyclodextrin (PA-β-CD). PA-β-CD generally provided higher Rs, whereas IPA-β-CD resulted in shorter migration times. A D-optimal design of experiment (DoE) was used to evaluate the effects of CS type and concentration and ammonium acetate concentration. Under optimized conditions, Rs ranged from 1.4 for ketoprofen to 8.2 for suprofen [38].

François et al. evaluated two chiral ILs, ethylcholine bis(trifluoromethylsulfonyl)imide (EtChol NTf_2_) and phenylcholine bis(trifluoromethylsulfonyl)imide (PhChol NTf_2_), for the CE enantioseparation of six profens (carprofen, ibuprofen, indoprofen, ketoprofen, naproxen, and suprofen). Since the ILs alone showed no enantioselectivity, they were combined with heptakis(2,6-di-O-methyl)-β-cyclodextrin (Di-OMe-β-CD) or Tri-OMe-β-CD. In several cases, IL addition increased both effective enantioselectivity (α) and Rs, indicating synergistic interactions between the IL and CD. However, part of the increase in Rs was attributed to changes in EOF caused by the higher ionic strength of the BGE. Nine IL-CD-analyte combinations showed increases in both α and Rs beyond those produced by increasing ionic strength alone, supporting a genuine synergistic effect. Naproxen was not enantioseparated under any of the investigated conditions, while suprofen showed no enantioselectivity with Di-OMe-β-CD [39].

Prior to investigating chiral ILs for profen enantioseparation, François et al. examined the effect of the achiral imidazolium-based IL 1-butyl-3-methylimidazolium bis(trifluoromethanesulfonyl)imide (BMIM NTf_2_) on the electrophoretic behavior of carprofen, ketoprofen, naproxen, and suprofen in NACE. Two competing mechanisms were identified: interaction of the profens with BMIM cations adsorbed onto the capillary wall at lower IL concentrations and ion-pair formation with free BMIM cations in the BGE at higher concentrations [40].

**Figure 3:**
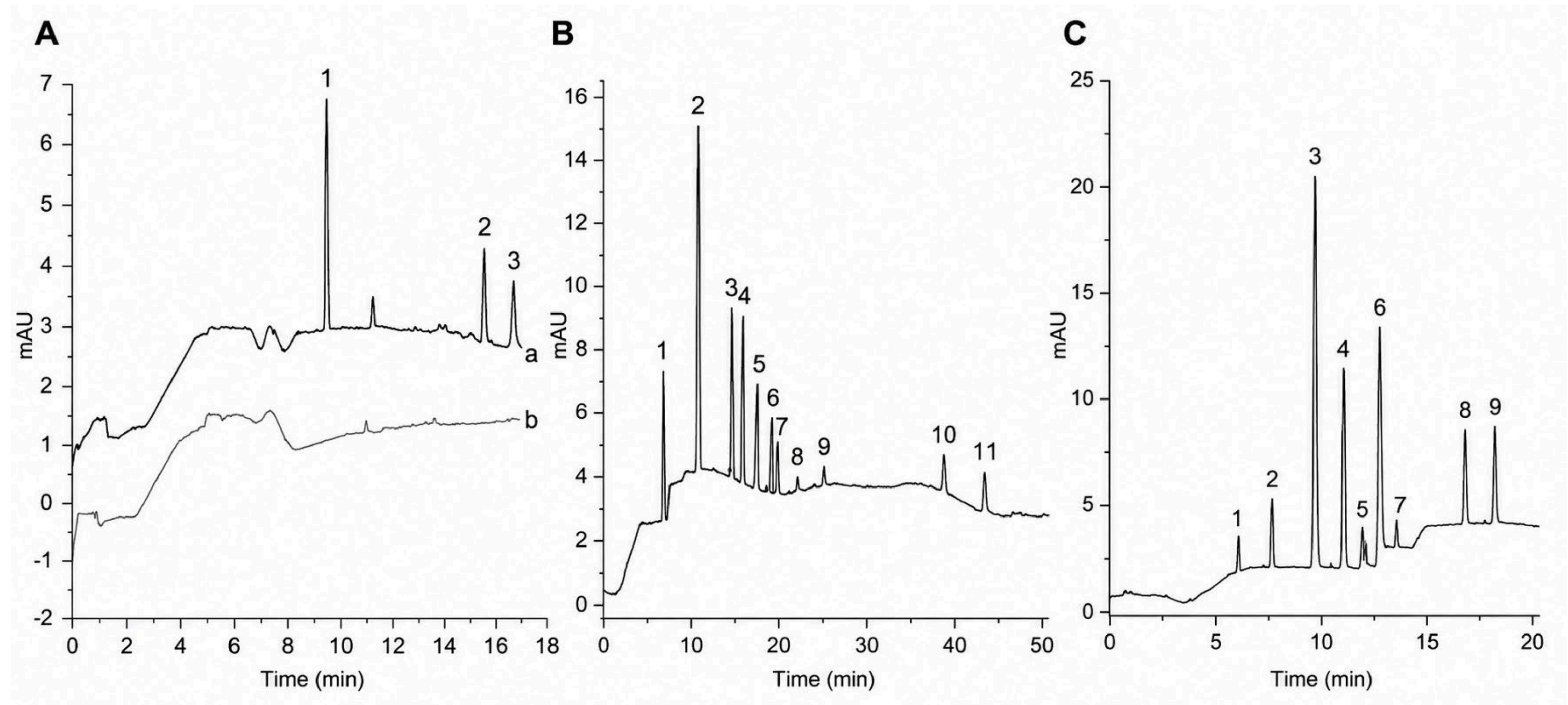
Representative NACE separations of profens and related NSAIDs. (**A**) Analysis of flurbiprofen enantiomers in human plasma following offline SPE: (a) spiked plasma and (b) blank plasma; peaks: 1, flufenamic acid (i.s); 2, *S*-flurbiprofen; 3, *R*-flurbiprofen. (**B**) Simultaneous enantioseparation of several profens; peaks: 1, flufenamic acid (i.s.); 2, ketoprofen enantiomers; 3-4, *S*- and *R*-flurbiprofen; 5, tiaprofenic acid enantiomers; 6-7, *S*- and *R*-suprofen; 8-9, *S*- and *R*-ibuprofen; 10-11, *S*- and *R*-indoprofen. (**C**) Separation of NSAIDs from different pharmacological classes, including *S*- and *R*-flurbiprofen (peaks 8-9). Adapted from Rousseau et al. [41] with permission from Wiley.

Rousseau et al. developed and validated a NACE method for the quantitative determination of flurbiprofen enantiomers in human plasma. Enantioseparation was achieved using PA-β-CD as CS in a methanolic BGE. Plasma samples were processed by SPE using Oasis MAX cartridges, with flufenamic acid as internal standard. The method provided an Rs of 4.8 within 20 min and was linear over 0.2–40 μg/mL for both enantiomers. LOD and LOQ were 0.06 and 0.20 μg/mL, respectively, while SPE recoveries reached 99% for *S*-flurbiprofen and 98% for *R*- flurbiprofen. Representative electropherograms obtained under these conditions, including the analysis of flurbiprofen enantiomers in human plasma and the simultaneous separation of several profens, are presented in Figure 3 [41].

Prokhorova et al. evaluated eremomycin, a macrocyclic glycopeptide antibiotic structurally related to vancomycin, as CS for the CE enantioseparation of fenoprofen, flurbiprofen, ibuprofen, indoprofen, and ketoprofen. The effects of BGE pH, eremomycin concentration, capillary dimensions, applied voltage, and organic modifiers on Rs were investigated. A phosphate BGE at pH 6.5 containing 2.5 mM eremomycin provided baseline enantioseparation of all five profens. The highest stereoselectivity was observed for flurbiprofen and indoprofen, while migration order studies for ibuprofen and ketoprofen showed faster migration of the *R*-enantiomer distomer [42].

Prokhorova et al. extended their previous work with eremomycin by using a chitosan-coated capillary to reduce adsorption of the CS onto the capillary wall. The approach was evaluated for several aromatic carboxylic acids, including fenoprofen, flurbiprofen, ibuprofen, indoprofen, and ketoprofen. The chitosan coating reversed the EOF and allowed lower BGE and eremomycin concentrations to be used without external pressure. Optimal conditions varied among the profens, with 0.75–1.6 mM eremomycin in 20 mM acetate BGE at pH 4.5–6.2. Compared with the uncoated capillary, migration times were reduced to less than 7 min and separation efficiency increased, although at the expense of stereoselectivity [43].

Further work by the same research group addressed eremomycin adsorption onto the fused-silica capillary wall. Lebedeva et al. investigated a water-MeOH BGE as a simpler alternative to capillary coating. MeOH reduced eremomycin adsorption and Joule heating while improving baseline stability and repeatability. Under optimized conditions (50 mM phosphate BGE, pH 5.8, 60% MeOH, 2 mM eremomycin), baseline enantioseparation was achieved for fenoprofen, flurbiprofen, indoprofen, and ketoprofen, with Rs values of 2.01, 5.92, 4.65, and 3.80, respectively. Compared with the chitosan-coated capillary, the water-MeOH system provided a technically simpler approach while maintaining effective enantioseparation within 15 min. The method was also applied to the determination of ketoprofen in a pharmaceutical gel [44].

A different approach to profen enantioseparation was reported by Riesová et al., who combined affinity CE with theoretical modeling. Acid dissociation and complexation constants for flurbiprofen, ibuprofen, ketoprofen, and naproxen with β-CD and Tri-OMe-β-CD were experimentally determined, with pKa values corrected to thermodynamic constants, and used as input parameters for Simul 5 Complex. The simulations accurately reproduced experimental migration behavior, including electromigration dispersion and migration-order reversals, and were subsequently used to optimize the enantioseparation conditions [45].

Cucinotta et al. investigated the lysine-bridged hemispherodextrin THLYSH as CS for the CE enantioseparation of flurbiprofen, ibuprofen, suprofen, and tiaprofenic acid. THLYSH is a trehalose-capped β-CD derivative containing ionizable lysine bridges, allowing its charge to be controlled by BGE pH. At pH 6.2, the negatively charged profens interacted with positively charged THLYSH through a combination of inclusion and electrostatic interactions. Using 1 mM THLYSH, all four racemates could be separated simultaneously, with Rs of 1.69 for flurbiprofen and 4.23 for tiaprofenic acid, while only partial enantioseparation was obtained for ibuprofen and suprofen. In contrast to the hemispherodextrins investigated previously by the same group, THLYSH could be used as a single CS without requiring a binary selector system [46].

Liu et al. developed an open-tubular capillary electrochromatography (OT-CEC) system using a graphene oxide (GO)-immobilized polymer-coated capillary for the enantioseparation of naproxen and pranoprofen, together with warfarin. Methyl-β-cyclodextrin (Me-β-CD) was used as CS. In a bare fused-silica capillary, Me-β-CD did not provide enantioseparation of any of the three analytes, whereas the GO-modified capillary enabled baseline separation. This improvement was attributed to adsorption of Me-β-CD onto the GO surface, increasing analyte–CS interactions and modifying the chiral recognition environment. Molecular docking supported this mechanism, showing larger differences in binding energies between the enantiomers in the GO/Me-β-CD system [47].

Liu et al. proposed a combined capillary electrophoresis–circular dichroism (CE–CD) approach for enantiomer identification and quantification without individual optically pure standards. The approach was evaluated for seven chiral compounds, including ibuprofen and naproxen. After enantioseparation by CE, the configuration of the predominant enantiomer in nonracemic mixtures was assigned by comparing experimental CD spectra with theoretical spectra calculated by time-dependent density functional theory (TDDFT). The assigned enantiomer was then correlated with the larger CE peak, enabling peak identification and quantification. For ibuprofen, used to evaluate quantitative performance, deviations between calculated and actual concentrations ranged from −1.8 to +3.4% [48].

Naghdi et al. developed a vancomycin-mediated CE method for the enantioseparation and quantitative determination of ibuprofen, combined with MoS_2_-based dispersive solid-phase extraction (DSPE) for sample cleanup and preconcentration. The CE and extraction conditions were optimized using a central composite design (CCD). Under optimized conditions (100 mM phosphate BGE, pH 6.5, 1 mM vancomycin), baseline enantioseparation was obtained. The method showed a linear range of 0.125–5.0 μg/mL, with LOD and LOQ values of 0.025 and 0.070 μg/mL, respectively, while DSPE provided a preconcentration factor of 33.4. The method was applied to ibuprofen tablets and, following DSPE, to urban water and human urine samples [49].

Kravchenko et al. developed a multifunctional covalent capillary coating based on imidazole and β-CD, with immobilized β-CD moieties acting as chiral recognition sites. The approach enabled the enantioseparation of ketoprofen without addition of a CS to the BGE. Coatings prepared using 500 mg/mL Ts-β-CD provided insufficient chiral discrimination, whereas increasing the concentration to 750 mg/mL, together with a higher silylating agent concentration, resulted in baseline enantioseparation (α = 1.03). The covalent coating also showed improved stability and repeatability compared with analogous dynamic coatings [50].

**Figure 4:**
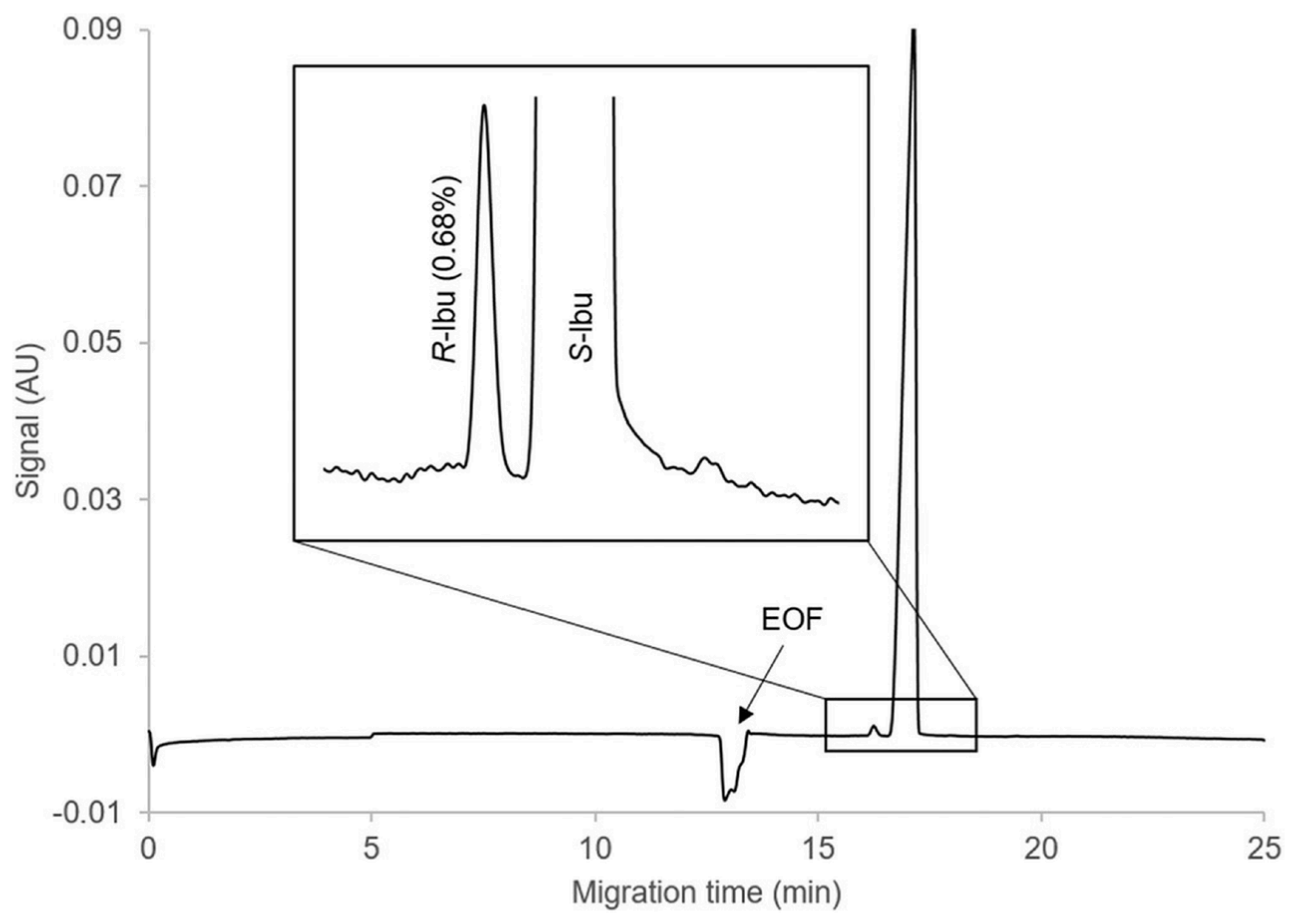
Electropherogram of an *S*-ibuprofen sample (0.6 mg/mL) containing 0.68% *R*-ibuprofen. Electrophoretic conditions: uncoated fused-silica capillary, 60.2 cm × 75 μm i.d. (50.0 cm effective length), 50 mM sodium acetate BGE, pH 5.0, 35 mM Tri-OMe-β-CD, 10 mM magnesium acetate; +26 kV; 27 °C; hydrodynamic injection, 3.45 kPa for 5 s; DAD detection at 202 nm. Adapted from Urlaub et al. [51] with permission from Wiley.

Urlaub et al. developed a chiral CE method for the determination of *R*-ibuprofen as an enantiomeric impurity in dexibuprofen (*S*-ibuprofen). Tri-OMe-β-CD was used as CS, while the addition of Mg^2+^ to the BGE substantially improved Rs. Under optimized conditions (50 mM sodium acetate BGE, pH 5.0, 35 mM Tri-OMe-β-CD, 10 mM magnesium acetate), Rs ≥ 2.3 was achieved within approximately 18 min, with *R*-ibuprofen migrating before *S*-ibuprofen. The method was validated according to ICH Q2(R1) and applied to investigate the configurational stability of dexibuprofen under mechanical stress induced by ball milling. Isomerization occurred mainly under basic conditions in the presence of KOH and increased with milling time and frequency. A representative electropherogram illustrating the determination of *R*-ibuprofen as an enantiomeric impurity is shown in Figure 4 [51].

Naghdi & Fakhari compared vancomycin, HP-β-CD, and *L*-tryptophan as CSs for the CE enantioseparation of naproxen. Vancomycin provided baseline enantioseparation at considerably lower concentrations than HP-β-CD, whereas *L*-tryptophan was ineffective as a BGE additive. The optimized vancomycin system used 0.5 mM CS in phosphate BGE at pH 6.0, while the HP-β-CD system required 7.71 mg/mL CS at pH 5.0. Thermodynamic analysis indicated that enantioseparation with HP-β-CD was predominantly enthalpy-driven, whereas both enthalpic and entropic contributions were involved with vancomycin, with electrostatic interactions playing an important role in chiral recognition [52].

Sun et al. developed a biphasic chiral recognition system in CEC for the enantioseparation of carprofen, flurbiprofen, ibuprofen, ketoprofen, and loxoprofen. The system combined HP-β-CD as a mobile-phase CS with a mixed bovine serum albumin (BSA)/pepsin monolithic CSP, providing chiral recognition in both the mobile and stationary phases. Among the β-CD derivatives tested, HP-β-CD provided the highest enantioselectivity. Under optimized conditions (0.05 M HP-β-CD in 10 mM phosphate BGE at pH 7.0), Rs values of 5.09, 6.01, 4.48, 5.65, and 4.26 were obtained for carprofen, flurbiprofen, ibuprofen, ketoprofen, and loxoprofen, respectively. The biphasic system provided substantially higher Rs than either the mobile-phase CS or CSP used individually, indicating a cooperative effect between the two chiral recognition mechanisms [53].

Zheng et al. developed a Metal–Organic Framework (MOF)-based CSP for OT-CEC using {(HQA)(ZnCl_2_)(2.5H_2_O)}_n_, prepared from 6-methoxyl-(8*S*,9*R*)-cinchonan-9-ol-3-carboxylic acid and ZnCl_2_ and immobilized on the inner wall of a fused-silica capillary by an in situ growth approach. The chiral separation performance of the MOF-coated capillary was evaluated using a broad range of structurally diverse chiral compounds, including 19 amino acids and the drugs chlorpheniramine, ibuprofen, and metoprolol. Baseline or partial enantioseparation was obtained for most of the investigated analytes. For ibuprofen, under optimized conditions (20 mM phosphate BGE, pH 6.75, 10% MeOH, +15 kV, 25 °C), an Rs of 2.71 was achieved, with migration times of 15.45 and 16.73 min, whereas no enantioseparation was observed using the bare capillary. The authors attributed chiral recognition to the porous structure and multiple interaction sites of the MOF, involving ion-pairing, hydrogen-bonding, dipole–dipole, π–π, and steric interactions [54].

Yun et al. developed a CE enantioseparation system using BSA-modified gold nanoparticles (BSA-AuNPs) as a quasi-stationary phase (QSP) for six profens (fenoprofen, flurbiprofen, ibuprofen, indoprofen, ketoprofen, and pranoprofen). Immobilization of BSA on gold nanoparticles (AuNPs) markedly enhanced chiral recognition compared with free BSA, increasing Rs from 0–0.89 to 5.34–15.21 and providing baseline enantioseparation of all six profens. Optimal conditions included 15 mM phosphate BGE at pH 8.0, 20 mM BSA, 3.2 nM AuNPs. Molecular docking showed a positive relationship between differences in BSA-enantiomer binding energies and experimental Rs, with fenoprofen exhibiting both the largest binding-energy difference and the highest Rs [55].

Kolobova et al. investigated physically adsorbed chitosan coatings and their contribution to chiral recognition in dual-selector CE systems. For profens, chitosan alone, either as a capillary coating or BGE additive, did not provide enantioseparation. However, combining a chitosan-coated capillary with vancomycin markedly improved the enantioseparation of ketoprofen. Under optimized conditions (25 mM phosphate BGE, pH 4.2, 2.5 mM vancomycin, 10% MeOH), Rs increased from 1.4 in the unmodified capillary to 3.0 with the chitosan coating. These results indicate a cooperative effect between the chitosan coating and vancomycin rather than independent chiral recognition by chitosan [56].

Tang et al. developed a lipase-based CSP for OT-CEC by encapsulating porcine pancreatic lipase within the MOF HKUST-1 (Lipase@HKUST-1). The composite material was prepared using a one-pot in situ encapsulation strategy and subsequently grown directly inside the capillary, with the MOF providing stable support for the enzyme while preserving its inherent chiral recognition ability. The Lipase@HKUST-1 stationary phase was evaluated for the enantioseparation of structurally different chiral compounds, including amino acids, chiral alcohols, and chiral drugs. Among the latter, successful enantioseparation of naproxen and chlorpheniramine was achieved. Theoretical calculations were additionally used to investigate the interactions involved in the chiral recognition process. This approach demonstrates the potential of combining the high porosity and stability of MOFs with the intrinsic stereoselectivity of enzymes for the development of novel chiral stationary phases in CEC [57].

The main characteristics of the reported CE methods for profen enantioseparation, including the investigated analytes, sample matrices, separation conditions, and analytical performance, are summarized in Table 2.

## 3. Discussion

CDs are by far the most frequently used CSs for the CE enantioseparation of profens, with β-CD derivatives accounting for most of the reported applications. Methylated derivatives, particularly Tri-OMe-β-CD, have shown high applicability, providing baseline separation for numerous profens under both aqueous and nonaqueous conditions. However, their performance varies considerably among individual analytes. Even closely related profens can exhibit markedly different Rs values under identical analytical conditions, and a CS providing excellent resolution for one compound may produce only partial or no separation for another. Such differences show that the fit of the aromatic moiety within the CD cavity alone does not adequately explain profen enantiodiscrimination. The extent and orientation of inclusion, interactions of the carboxylate group and aromatic substituents with the CD rim, and the substitution pattern of the CD all contribute to the stability and mobility of the resulting transient complexes [13]. BGE pH is particularly important for profens because it determines their ionization state and, together with the charge of the CS, affects both complexation and electrophoretic mobility. Organic modifiers can further alter hydrophobic interactions, CD complexation and EOF, explaining why changes in BGE composition sometimes produce substantial improvements in Rs [58].

The limitations of single-selector systems prompted the use of combinations of CDs with different physicochemical properties. Dual-selector systems were particularly effective when a neutral CD responsible mainly for stereoselective complexation was combined with a charged CD capable of introducing an additional mobility difference between the enantiomer-selector complexes [59]. The magnitude of the improvement can be substantial: several studies summarized in Table 2 reported Rs values considerably higher than those obtained with the corresponding single-selector systems. However, this effect is not universal and depends on the analyte–selector combination. Moreover, dual systems introduce additional variables, including the concentration and ratio of the two selectors, BGE pH and ionic strength, which can interact and make optimization difficult [59]. The application of mathematical models and DoE in several studies is therefore particularly relevant, as these approaches permit the simultaneous evaluation of CS interactions rather than optimization of each parameter independently [11].

Alternative CSs have provided useful solutions when CD-mediated recognition was insufficient. Macrocyclic antibiotics, particularly eremomycin and vancomycin, produced good enantioselectivity for several profens, although adsorption of these large molecules onto fused-silica surfaces can impair current stability and migration-time repeatability. The studies using eremomycin illustrate two practical solutions to this problem: modification of the capillary surface and the use of hydro-organic BGEs to reduce selector adsorption. The latter approach is analytically attractive because it avoids permanent capillary modification while retaining high Rs. Protein-based systems represent another effective recognition mechanism. BSA, either in solution or incorporated into monolithic stationary phases, afforded high Rs values for several profens, and the addition of AuNPs further enhanced the separations reported with BSA. These systems demonstrate that high profen enantioselectivity is not restricted to inclusion-type recognition by CDs, although their higher experimental complexity may limit their transfer to routine analysis.

The compound-dependent behavior of profens reflects the complexity of chiral recognition. In CD-based systems, enantioselectivity results from a combination of hydrophobic inclusion, hydrogen bonding, electrostatic, and steric interactions. Differences in aromatic substitution and hydrophobicity can modify the geometry and stability of the resulting diastereomeric complexes, explaining why the same CD may provide markedly different selectivity among profens. CD substitution further modifies these interactions, while charged CDs also influence electrophoretic mobility. Alternative selectors, such as macrocyclic antibiotics and proteins, offer additional stereoselective interactions but their routine use may be limited by capillary adsorption, UV absorption, stability, and more demanding optimization.

Capillary surface modification represents a recurring strategy in the reviewed literature. PVA-, polybrene- and chitosan-coated capillaries were used to suppress or reverse EOF, reduce undesirable wall interactions, or create favorable conditions for a particular CS. Covalently immobilized CD phases and CEC approaches went a step further by incorporating the chiral recognition element into the stationary phase. These configurations reduce or eliminate the need for high concentrations of soluble CS and may improve system stability, but preparation and reproducibility of the modified capillary become additional analytical variables. The combination of stationary and mobile CSs, as demonstrated with mixed protein monolith/CD systems, offers another means of increasing selectivity through complementary recognition mechanisms.

NACE constitutes a distinct approach within the profen enantioseparation techniques. Methanolic BGEs containing derivatized CDs provided effective separations for several compounds and, in some cases, enabled direct application to biological samples after SPE. Changing from water to an organic medium modifies analyte solvation, acid–base behavior, selector–analyte interactions and electrophoretic mobility, and can therefore produce selectivity that is difficult to obtain in aqueous BGEs [60]. The successful determination of flurbiprofen enantiomers in plasma and urine illustrates the analytical potential of this approach beyond CS screening. Nevertheless, the need to optimize the CS separately for different profens again emphasizes the strong analyte dependence of chiral recognition within this structurally related class.

A clear distinction should also be made between studies aimed primarily at demonstrating enantioseparation and methods developed for quantitative analysis. A large proportion of the literature summarized in Table 2 reports Rs or selectivity values using standard solutions, with little or no analytical validation. A smaller group of studies addressed specific analytical problems, including determination of *R*-ibuprofen in *S*-ibuprofen, measurement of enantiomeric impurities in single-enantiomer pharmaceutical products, investigation of configurational stability, pharmacokinetic analysis of ketoprofen enantiomers, and determination of profens in environmental samples. These applications are particularly relevant because they demonstrate that high Rs alone is not sufficient for practical chiral analysis. Sensitivity, precision, recovery, robustness and the ability to quantify a distomer in the presence of the eutomer become equally important.

From a quantitative perspective, the reported CE methods show substantial analyte- and application-dependent variability. For the most extensively investigated profens, baseline or substantially higher enantiomeric resolution has frequently been achieved, with reported Rs values reaching 12.5 for ibuprofen and 6.5 for flurbiprofen using oppositely charged dual-CD systems [33], 6.1 for ketoprofen using an HS-β-CD/Tri-OMe-β-CD system [29], and 8.0 for naproxen using a dual CM-β-CD/Tri-OMe-β-CD system under reversed polarity [34]. In validated bioanalytical applications using conventional UV detection, LOD values for ibuprofen, flurbiprofen, and ketoprofen were generally in the sub-μg/mL range. A stereospecific CZE method for human serum provided LOD values of 0.25 μg/mL for flurbiprofen and ketoprofen and 0.50 μg/mL for ibuprofen [36], while an SPE-based method for ibuprofen achieved LOD/LOQ values of 0.05/0.10 μg/mL in serum and 0.25/0.50 μg/mL in urine [37].

The practical relevance of enantioseparation is particularly evident for single-enantiomer profen products, in which the distomer represents a critical stereochemical quality attribute. Several of the reviewed methods directly address this requirement. For *S*-ketoprofen, Blanco et al. developed and validated a CE method that achieved a relative LOD of 0.04% for *R*-ketoprofen and detected R-ketoprofen impurity levels between 0.1 and 0.4% in pharmaceutical stability samples [32]. For ibuprofen, Simó et al. demonstrated detection of 0.5% *R*-ibuprofen in the presence of 99.5% *S*-ibuprofen [31], while La et al. similarly demonstrated detection of a 0.5% *R*-enantiomeric impurity using CD-modified CE [34]. More recently, Urlaub et al. developed a method specifically for determination of *R*-ibuprofen as an enantiomeric impurity in dexibuprofen, achieving Rs ≥ 2.3 and validating the method according to ICH Q2(R1) [51].

Limited concentration sensitivity remains an important practical constraint for many of the reported CE-UV methods. UV detection is used in most studies and is adequate for pharmaceutical preparations and concentrated standards but becomes restrictive when profen enantiomers must be determined at low concentrations in biological or environmental matrices [61]. SPE and other preconcentration procedures have partly compensated for this limitation, allowing sub µg mL^−1^ detection in several applications, but at the expense of additional sample-preparation steps. Despite the extensive methodological work devoted to profen enantioseparation, relatively few studies combine high enantioselectivity, full quantitative validation and application to complex real samples. This discrepancy between excellent Rs and comparatively limited quantitative application remains one of the most evident gaps in the field.

Beyond detector sensitivity, the application of chiral CE to bioanalysis is limited by matrix compatibility and sample preparation, as proteins, salts, and endogenous components may interfere with separation and method reproducibility. Extraction and preconcentration strategies, particularly SPE, can reduce matrix effects while improving detectability [61]. CE–MS could substantially enhance sensitivity and selectivity, but remains underutilized in profen enantioanalysis, partly because nonvolatile BGEs, surfactants, and high concentrations of CSs may be incompatible with MS detection. Future developments should therefore combine MS-compatible separation conditions with efficient sample preparation and preconcentration strategies to facilitate quantitative analysis in complex biological matrices [62].

The profen literature shows that CE enantioseparation has progressed from relatively simple screening of individual CDs toward systems in which several sources of selectivity are deliberately combined or controlled. CDs remain the most practical and extensively investigated selectors, but dual-CD systems, macrocyclic antibiotics, capillary coatings, nonaqueous media, protein-based phases and nanoparticle-assisted systems have demonstrated that difficult separations can often be addressed by modifying either the recognition mechanism or the electrophoretic environment. At the same time, the available results do not support the existence of a generally applicable CS or set of conditions for the profen class. Method development therefore remains compound-specific, and the principal challenge is no longer simply to obtain baseline enantioseparation, but to translate this selectivity into robust, sensitive and validated methods suitable for pharmaceutical, biological and environmental analysis.

**Figure 5:**
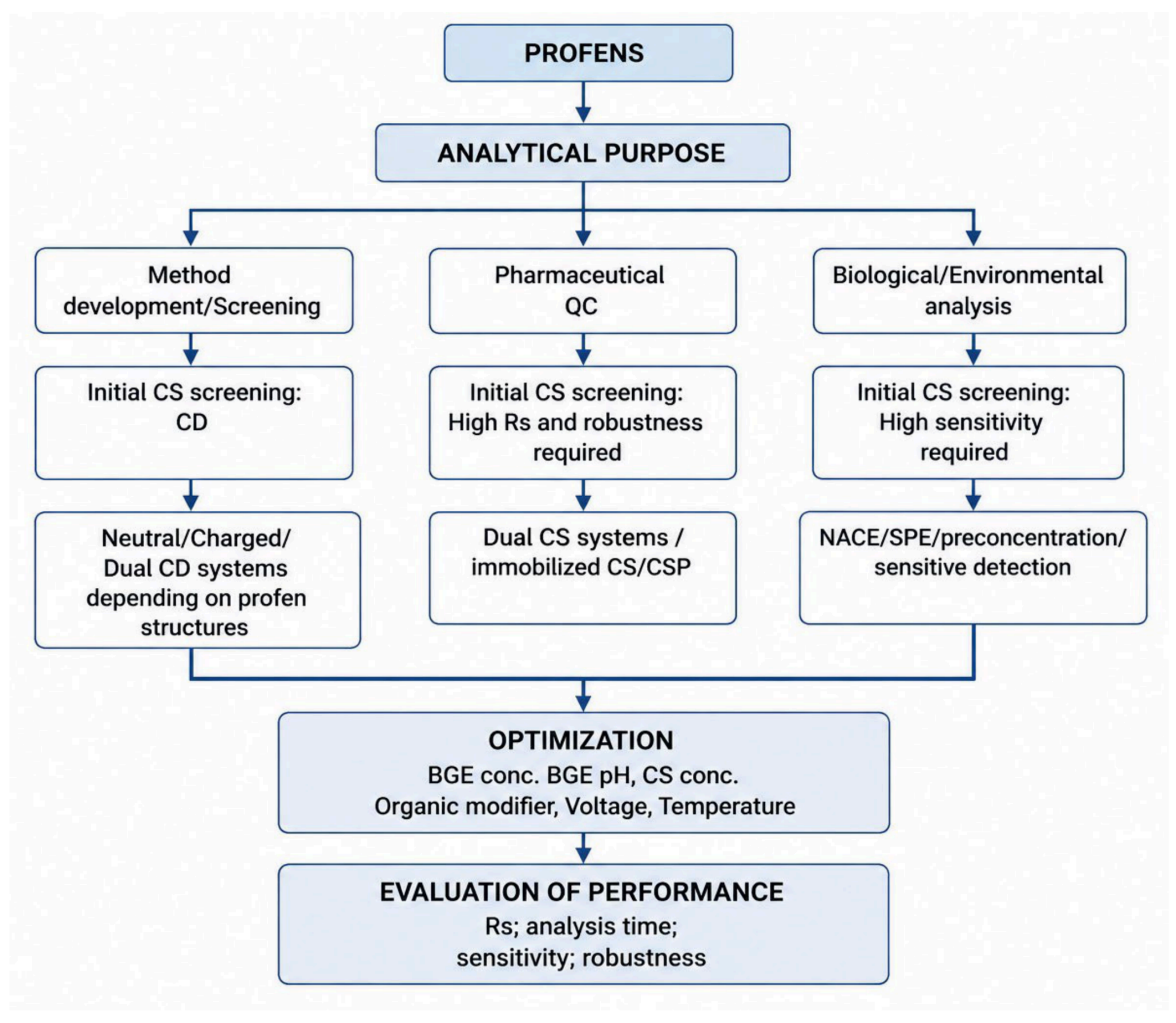
Decision-making workflow for chiral selector selection and method optimization in CE enantioseparation of profens.

Based on these considerations, a conceptual workflow for selecting an appropriate chiral separation strategy according to the analytical purpose and subsequent method optimization is proposed in Figure 5.

## 4. Materials and Methods

A structured literature search was conducted to identify publications describing the enantioseparation and enantioselective determination of profens by CE techniques. PubMed, Scopus, Web of Science, and Google Scholar were searched for publications available up to August 2026.

The search strategy combined terms related to CE (“capillary electrophoresis”, “CE”, “capillary zone electrophoresis”, “CZE”, “nonaqueous capillary electrophoresis”, “NACE”, “capillary electrochromatography”, and “CEC”) with terms related to chiral analysis (“chiral”, “enantioseparation”, “enantiomer”, “enantiomeric separation”, and “chiral selector”). Searches were additionally performed using the individual names of the profens considered in this review, including carprofen, fenoprofen, flurbiprofen, ibuprofen, indoprofen, ketoprofen, naproxen, pranoprofen, suprofen, and tiaprofenic acid. Reference lists of the retrieved publications and relevant review articles were manually examined to identify additional eligible studies.

Original research articles reporting the enantioseparation of at least one profen by CE or a related electromigration technique were considered eligible. Studies dealing exclusively with achiral analysis, separation techniques without an electrophoretic component, or compounds outside the scope of the review were excluded. Conference abstracts, conference proceedings without a full research article, reviews, book chapters, and other publications not providing sufficient experimental information for evaluation of the separation conditions were not included as primary studies. No restrictions were applied regarding the type of CS, electrophoretic mode, or sample matrix. Studies conducted using standard solutions as well as pharmaceutical, biological, environmental, and other matrices were considered.

For each eligible publication, data were extracted on the analytes, sample matrix, electrophoretic mode, capillary dimensions and surface modification, BGE composition and pH, type and concentration of the CS, applied voltage, temperature, injection conditions, and detection method. Where available, analytical and enantioseparation performance parameters, including Rs, α, analysis time, linear range, LOD and LOQ, precision, accuracy, recovery, and the lowest quantified enantiomeric impurity level, were also recorded.

Following screening for relevance and eligibility, 38 original research articles were included in the detailed chronological analysis and comparative summary presented in Table 2. Additional publications, including reviews and methodological papers, were used only to provide background, methodological context, and interpretation and were not considered primary studies. Because the search was designed to support a narrative rather than a systematic review, a formal PRISMA-based selection procedure and prospective record of the number of records retrieved and excluded at each screening stage were not performed. This represents a limitation of the present review and should be considered when interpreting the comprehensiveness of the literature coverage.

## 5. Conclusions

CE has proved to be a versatile approach for the enantioseparation of profens, accommodating compounds with different structural and stereochemical characteristics and allowing the use of a broad range of chiral recognition strategies. The accumulated literature also shows a transition from methods primarily focused on demonstrating enantioseparation toward quantitative applications addressing enantiomeric purity, pharmaceutical quality control, and the analysis of biological and environmental samples.

Despite these advances, several practical bottlenecks continue to limit the broader application of CE to profen enantioanalysis. These include the predominance of UV detection and its limited concentration sensitivity, the relatively small number of fully validated quantitative methods applied to complex samples, and the strongly compound-specific nature of CS selection and method optimization. Future studies should therefore focus on coupling CE with more sensitive and selective detection systems, particularly MS. Greater use of QbD/DoE strategies and molecular modeling could facilitate more rational CS selection and multivariate optimization, reducing reliance on empirical screening. Further validation in pharmaceutical quality control and expansion toward clinically and environmentally relevant matrices are also needed to demonstrate the practical transferability of CE enantioseparations. Addressing these limitations would help move the field from highly efficient proof-of-concept separations toward robust and broadly applicable methods for pharmaceutical, bioanalytical, and environmental analysis.

## Figures and Tables

**Table 1:** Chemical and stereochemical characteristics of representative profens.

Profen	Chemical Name	Stereochemical Form	Pharmaceutical/Clinical Status	Relevant Stereochemical Features
Carprofen	6-chloro-α-methyl-9H-carbazole-2-acetic acid	Racemate	Mainly veterinary use	Contains one stereogenic center; enantiomers show stereoselective pharmacological and metabolic behavior
Fenoprofen	2-[3-(phenoxy)phenyl]propanoic acid	Racemate	Marketed as racemic fenoprofen	Anti-inflammatory activity is predominantly associated with the *S*-enantiomer
Flurbiprofen	2-(2-fluoro-4-biphenylyl)propanoic acid	Racemate	Marketed as racemic flurbiprofen	*S*-enantiomer is the stronger COX inhibitor; stereoselective pharmacological effects have been reported
Ibuprofen	2-[4-(2-methylpropyl)phenyl]propanoic acid	Racemate/*S*-enantiomer	Ibuprofen marketed as racemate; *S*-ibuprofen marketed as dexibuprofen	Chiral-switch example; substantial unidirectional metabolic inversion of *R*- to *S*-ibuprofen
Ketoprofen	2-(3-benzoylphenyl)propanoic acid	Racemate/*S*-enantiomer	Ketoprofen marketed as racemate; *S*-ketoprofen marketed as dexketoprofen	Classical chiral-switch example; metabolic inversion of *R*- to *S*-ketoprofen occurs to a substantially smaller extent than for ibuprofen
Naproxen	2-(6-methoxynaphthalen-2-yl)propanoic acid	*S*-enantiomer	Used therapeutically as *S*-naproxen	*S*-enantiomer accounts for the desired anti-inflammatory activity; *R*-naproxen has been associated with hepatotoxicity
Pranoprofen	2-(5H-[1]benzopyrano[2,3-b]pyridin-7-yl)propanoic acid	Racemate	Used as an NSAID in ophthalmic therapy	Contains one stereogenic center
Suprofen	2-[4-(2-thienylcarbonyl)phenyl]propanoic acid	Racemate	Historically marketed as racemic suprofen	Contains one stereogenic center
Tiaprofenic acid	2-[5-(benzoyl)-2-thienyl]propanoic acid	Racemate	Marketed as racemic tiaprofenic acid	Contains one stereogenic center

**Table 2:** CE methods for the enantioseparation and analysis of profens.

CE Method/CS	Analytes	Matrix	CE Conditions	Analytical Performance	Reference
CZE Maltodextrin, maltooligosaccharide mixtures	Flurbiprofen, Ibuprofen, Ketoprofen	Standard solutions	Fused-silica capillary, 50–90 cm × 50 or 75 μm i.d.; 10 mM sodium phosphate BGE, pH 7.0; +30 kV; UV, 214 nm	Baseline separation obtained for ibuprofen under several conditions; flurbiprofen showed lower enantioseparation; ketoprofen was not separated with the maltodextrin mixtures tested. RCS used instead of conventional Rs	[20]
CGE HP-β-CD	Naproxen	Standard solutions; S-naproxen samples for enantiomeric purity assessment	Uncoated fused-silica capillary, 27 cm × 25 μm i.d. (20 cm effective length); 200 mM MES/TBAH BGE, pH 5.0, containing 10 mM HP-β-CD and 0.4% polymeric additive; reversed polarity; 700 V/cm; 20 °C; UV detection at 230 nm.	Rs = 3.54; α ≈ 1.095; linear range: 1 μg/mL–10 mg/mL; LOD: 300 nM for *R*-naproxen (≈8 × 10^−14^ g on-column); a 0.11% increase in *R*-naproxen impurity was detected (1.26–1.37%).	[21]
CZE-UV derivatized β-CDs	Ibuprofen, Fenoprofen, Flurbiprofen, Indoprofen, Ketoprofen, Suprofen; commercial ibuprofen and ketoprofen tablets	Standard solutions; pharmaceutical formulations	Polyacrylamide-coated fused-silica capillary, 35 cm × 50 μm i.d. (31.5 cm effective length); 100 mM MES BGE, pH 5.0, containing Tri-OMe-β-CD, Di-OMe-β-CD, or MeNH-β-CD (0–30 mM) and 0–40% (*v*/*v*) MeOH; +20 kV; 25 °C; pressure injection, 10 psi·s; UV detection at 206 nm.	Baseline enantioseparation of all six profens with Tri-OMe-β-CD; minimum CS concentrations for baseline separation: indoprofen <5 mM, fenoprofen and ibuprofen 7.5 mM, flurbiprofen, ketoprofen, and suprofen 20 mM. With 5 mM Tri-OMe-β-CD, addition of MeOH increased flurbiprofen Rs from <0.5 to 1.4 (20% MeOH) and 2.5 (40% MeOH).	[22]
CZE-UV derivatized CDs	Carprofen, Flurbiprofen, Indoprofen, Ketoprofen, Naproxen, Pranoprofen, Suprofen	Standard solutions	Uncoated fused-silica capillary, 60 cm × 50 μm i.d. (52.5 cm effective length); 75 mM BGE (formate, pH 4.0; MES, pH 6.0; bicine, pH 8.0; β-alanine, pH 10.0) containing β-CD, HP-β-CD, DM-β-CD, TM-β-CD, or HP-γ-CD (1–20 mM); +30 kV; 26 °C; hydrodynamic injection, 10–15 s; UV detection at 254 nm.	With 10 mM TM-β-CD at pH 4.0, baseline enantioseparation was obtained for carprofen (Rs = 2.2), indoprofen (Rs = 2.4), ketoprofen (Rs = 1.5), pranoprofen (Rs = 3.8), and suprofen (Rs = 1.9); flurbiprofen was only partially resolved (Rs = 0.7).	[23]
CZE-UV Dual-CD system (SBE-β-CD + TM-β-CD)	Carprofen, Fenoprofen, Flurbiprofen, Ibuprofen, Indoprofen, Ketoprofen, Suprofen, Tiaprofenic acid	Standard solutions	Uncoated fused-silica capillary, 44 cm × 50 μm i.d. (37 cm effective length); 100 mM phosphoric acid-triethanolamine BGE, pH 3.0, containing 5 mM SBE-β-CD and 10–40 mM TM-β-CD; reversed polarity, −25 kV; 25 °C; hydrodynamic injection, 5 s; UV detection at 210–280 nm.	Complete enantioseparation of all investigated profens, generally within 15 min; Rs = 30.6 for carprofen, 16.3 for fenoprofen, 16.3 for flurbiprofen, 5.6 for ibuprofen, 4.9 for indoprofen, 11.3 for ketoprofen, 8.9 for suprofen, and 1.3 for tiaprofenic acid.	[24]
CZE Tri-OMe-β-CD, Hepta-MeNH-β-CD, vancomycin (partial filling)	Carprofen, Cicloprofen, Flurbiprofen, Ibuprofen, Indoprofen, Ketoprofen, Naproxen, Suprofen	Standard solutions	Polyacrylamide-coated fused-silica capillary, 35 cm × 50 μm i.d. (30.5 cm effective length); 75 mM Britton–Robinson BGE, pH 5.0, containing 30 mM Tri-OMe-β-CD, 5 mM Hepta-MeNH-β-CD, or 5 mM vancomycin (partial filling); −15 to −20 kV; 25 °C; pressure injection, 10 psi·s; UV detection at 206 or 254 nm.	Vancomycin: Rs = 3.1 (carprofen), 4.2 (cicloprofen), 6.0 (flurbiprofen), 2.3 (ibuprofen), 1.6 (indoprofen), 5.0 (ketoprofen), 3.4 (naproxen), and 2.3 (suprofen). LOD: Tri-OMe-β-CD, 5 × 10^−6^ M (indoprofen, naproxen, suprofen); Hepta-MeNH-β-CD, 1 × 10^−5^ M (all analytes); vancomycin, 1 × 10^−6^ M (indoprofen) and 5 × 10^−6^ M (naproxen, suprofen).	[25]
NACE-UV QA-β-CD	Carprofen, Fenoprofen, Flurbiprofen, Ibuprofen, Indoprofen, Ketoprofen, Suprofen	Standard solutions; commercial ketoprofen tablets	Uncoated fused-silica capillary, 42 cm × 50 μm i.d. (31 cm effective length); 100 mM Tris–100 mM acetic acid BGE in formamide, pH 7.5, containing 2.57–4.28% (*w*/*v*) QA-β-CD (≈15–25 mM); −476 V/cm; 30 °C; gravity injection; UV detection at 254 nm.	Baseline enantioseparation of fenoprofen with ≈200,000 theoretical plates using 15 mM QA-β-CD; carprofen, flurbiprofen, indoprofen, ketoprofen, and suprofen were also resolved in formamide, whereas ibuprofen and naproxen showed no enantioseparation.	[26]
CZE-UV Tri-OMe-β-CD	Fenoprofen, Ibuprofen, Ketoprofen	Standard solutions	Uncoated fused-silica capillary, 64.5 cm × 50 μm i.d. (56 cm effective length); 20 mM phosphate-20 mM triethanolamine BGE, pH 5.0, containing 25 mM Tri-OMe-β-CD; +20 kV; 35 °C; hydrodynamic injection, 75 mbar·s; UV detection at 253 nm	Complete enantioseparation within 20 min; Rs = 4.76 for fenoprofen, 4.17 for ibuprofen, and 3.86 for ketoprofen.	[27]
CZE-UV QSER modeling (multivariate linear regression and neural networks)	Fenoprofen, Flurbiprofen, Ibuprofen, Ketoprofen, Suprofen; 17 other profens	Standard solutions	Uncoated fused-silica capillary, 64.5 cm × 50 μm i.d. (56 cm effective length); phosphate–citrate BGE, pH 5.0, containing 12 mM β-CD, 50 mM HP-β-CD, or 50 mM Tri-OMe-β-CD; +20 kV; 35 °C; hydrodynamic injection, 30 mbar for 3 s; UV detection at 200 nm (254 nm for the neutral marker).	Tri-OMe-β-CD provided the highest enantioselectivity: ξ = 1.233 for fenoprofen, 1.098 for flurbiprofen, 1.175 for ibuprofen, 1.074 for ketoprofen, and 1.084 for suprofen.	[28]
CZE-UV Dual-CD system (HS-β-CD + Tri-OMe-β-CD)	Fenoprofen, Flurbiprofen, Ibuprofen, Ketoprofen	Standard solutions	Uncoated fused-silica capillary, 45 cm × 50 μm i.d. (40.3 cm effective length); 100 mM phosphoric acid–triethanolamine BGE, pH 2.5, containing HS-β-CD/Tri-OMe-β-CD at 3/20 mM for fenoprofen, 3/10 mM for flurbiprofen and ibuprofen, and 2/20 mM for ketoprofen; reversed polarity, −25 kV; 25 °C; hydrodynamic injection, 5 s; UV detection at 214 nm	Complete enantioseparation with the optimized dual-CD systems; Rs = 4.3 for fenoprofen, 2.9 for flurbiprofen, 3.3 for ibuprofen, and 6.1 for ketoprofen. Predicted selectivities were in close agreement with experimental values, supporting the validity of the mathematical model.	[29]
EKC hemispherodextrins	Flurbiprofen, Ibuprofen, Indoprofen, Ketoprofen, Suprofen, Thiaprofenic acid	Standard solutions	Uncoated fused-silica capillary, 69 cm × 75 μm i.d. (59.2 cm effective length); 33 mM sodium acetate–33 mM boric acid BGE, pH 6.9, containing THALAH, THAMH, or THCMH (0.30–3.0 mM), individually or as binary mixtures (1.0 mM total CS; 3:1, 1:1, or 1:3 ratios); +25 kV; 20 °C; electrokinetic injection, 10 kV; DAD detection.	Best individual enantioseparations: Rs = 1.94 for flurbiprofen (2 mM THAMH), 1.38 for ibuprofen (3 mM THCMH), 2.45 for indoprofen (0.8 mM THCMH), 2.33 for ketoprofen (2 mM THAMH), 1.17 for suprofen (0.3 mM THCMH), and 1.35 for tiaprofenic acid (0.8 mM THAMH).	[30]
CZE-UV Dextrin 10	Ibuprofen	Polymeric drug-delivery systems; phosphate buffer; rat plasma	Uncoated fused-silica capillary, 37 cm × 50 μm i.d. (30 cm effective length); 150 mM sodium tetraborate BGE, pH 9.0, containing 6% (*w*/*v*) Dextrin 10; +20 kV; 20 °C; hydrodynamic injection, 0.5 psi for 1–3 s; UV detection at 200 nm.	Rs > 1.25; linear range: 1–40 μg/mL (r^2^ = 0.998–0.999); LOQ: 1 μg/mL; migration-time RSD ≤ 0.45%; peak-area RSD ≤ 3.09%; 0.5% *R*-ibuprofen was detectable in the presence of 99.5% *S*-ibuprofen.	[31]
CZE-UV Tri-OMe-β-CD	Ketoprofen	Oral pharmaceutical formulations; stability study samples	Extended light-path fused-silica capillary, 56 cm effective length × 50 μm i.d.; 40 mM acetate BGE, pH 5.0, containing 75 mM Tri-OMe-β-CD; +20 kV; 35 °C; hydrodynamic injection, 50 mbar for 5 s; UV detection at 198 nm.	Rs = 3.77; analysis time: 27 min; linear range: 1 × 10^−3^–2.5 × 10^−2^ mg/mL (R^2^ = 0.9998); LOD: 1 × 10^−3^ mg/mL; LOQ: 2 × 10^−3^ mg/mL; repeatability RSD: 8% (peak area) and 10% (migration time); intermediate precision RSD: 5% and 8%, respectively; recovery: 101%; relative LOD for the enantiomeric impurity: 0.04%.	[32]
CZE-UV Dual-CD system (HS-β-CD + PMMA-β-CD)	Fenoprofen, Flurbiprofen, Ibuprofen, Ketoprofen	Standard solutions	PVA-coated fused-silica capillary, 44 cm × 50 μm i.d. (37 cm effective length); 100 mM phosphoric acid–triethanolamine BGE, pH 2.5, containing 4 mM HS-β-CD and 18 mM PMMA-β-CD; reversed polarity, −25 kV; 25 °C; hydrodynamic injection, 7 s; UV detection at 214 nm.	Complete enantioseparation with the optimized dual-CD system; Rs = 11.4 for fenoprofen, 6.5 for flurbiprofen, 12.5 for ibuprofen, and 4.4 for ketoprofen; migration times: 12–21 min. Predicted selectivities were in good agreement with experimental values, supporting the mathematical model.	[33]
CZE-UV single- and dual-CD systems Tri-OMe-β-CD	Fenoprofen, Flurbiprofen, Ibuprofen, Indoprofen, Ketoprofen, Naproxen, Pirprofen, Suprofen, Tiaprofenic acid	Standard solutions	Uncoated fused-silica capillary, 50.2 cm × 50 μm i.d. (40 cm effective length); Normal polarity: 100 mM MES BGE, pH 6.0, containing 75 mM Tri-OMe-β-CD, +25 kV; Reverse polarity 100 mM phosphoric acid–triethanolamine BGE, pH 3.0, containing 30 mM Tri-OMe-β-CD, 1.0% CM-β-CD, and 0.01% HDB, +15 kV; UV detection at 200 nm.	Normal polarity: Rs = 3.9 for fenoprofen, 1.5 for flurbiprofen, 2.6 for ibuprofen, 1.0 for indoprofen, 1.8 for ketoprofen, 4.4 for naproxen, 2.7 for pirprofen, and 1.2 for suprofen; tiaprofenic acid, NR. Reverse polarity: Rs = 5.0 for fenoprofen, 4.8 for flurbiprofen, 4.6 for ibuprofen, 3.0 for ketoprofen, 8.0 for naproxen, 4.0 for pirprofen, 2.9 for suprofen, and 1.5 for tiaprofenic acid; Detection of 0.5% *R*-enantiomeric impurity was demonstrated.	[34]
MEKC cationic vesicles	Carprofen, Fenoprofen, Flurbiprofen, Ibuprofen, Indoprofen, Ketoprofen, Naproxen, Suprofen	Standard solutions	Uncoated fused-silica capillary, 60 cm × 50 μm i.d. (55.5 cm effective length); 20 mM phosphate BGE containing 10 mM DMEB, pH 7.4 for fenoprofen, flurbiprofen, ketoprofen, and naproxen, and pH 7.9 for carprofen, ibuprofen, indoprofen, and suprofen; −20 kV; 25 °C; UV detection at 254 nm.	ΔtR = 1.16 min for carprofen, 0.64 min for fenoprofen, 0.63 min for flurbiprofen, 0.98 min for ibuprofen, 1.49 min for indoprofen, 2.32 min for ketoprofen, 2.14 min for naproxen, and 1.53 min for suprofen. Simultaneous separation of mixtures containing up to five profens and their enantiomers was achieved in a single run	[35]
CZE-UV Tri-OMe-β-CD	Flurbiprofen, Ibuprofen, Ketoprofen (Naproxen used as I.S.)	Human serum	Uncoated fused-silica capillary, 60 cm × 75 μm i.d. (51.5 cm effective length); 20 mM triethanolamine–phosphate BGE, pH 5.0, containing 50 mM Tri-OMe-β-CD; +20 kV; 35 °C; hydrodynamic injection, 50 mbar for 5 s; UV detection at 253 nm.	Rs = 2.02 for flurbiprofen, 2.19 for ibuprofen, and 3.70 for ketoprofen; LOD: 0.25 μg/mL for flurbiprofen and ketoprofen and 0.50 μg/mL for ibuprofen; LOQ: 0.50 μg/mL for flurbiprofen and ketoprofen and 1.00 μg/mL for ibuprofen; recovery: 85–91% for ketoprofen (≈90% overall).	[36]
CZE-UV Tri-OMe-β-CD	Ibuprofen	Human serum and urine	Uncoated fused-silica capillary, 70 cm × 75 μm i.d. (61.5 cm effective length); 20 mM phosphate–triethanolamine BGE, pH 5.0, containing 50 mM Tri-OMe-β-CD; +25 kV; 30 °C; hydrodynamic injection, 50 mbar for 5 s; UV detection at 220 nm; C18 SPE for sample preparation.	Serum: Rs = 2.19; linear range: 0.10–25 μg/mL (r ≥ 0.9997); LOD/LOQ: 0.05/0.10 μg/mL; recovery: 74–86%. Urine: Rs = 2.25; linear range: 0.50–250 μg/mL (r ≥ 0.9991); LOD/LOQ: 0.25/0.50 μg/mL; recovery: 90–98%. Precision and accuracy: <15%.	[37]
NACE single-isomer amino-β-CDs	Fenoprofen, Flurbiprofen, Ibuprofen, Indoprofen, Ketoprofen, Suprofen, Tiaprofenic acid	Standard solutions	Uncoated fused-silica capillary, 48.5 cm × 75 μm i.d. (40 cm effective length); methanolic ammonium acetate BGE containing 5 mM IPA-β-CD and 40 mM ammonium acetate for ibuprofen and indoprofen; 10 mM PA-β-CD and 40 mM ammonium acetate for flurbiprofen, suprofen, and tiaprofenic acid; or 20 mM PA-β-CD and 20 mM ammonium acetate for fenoprofen and ketoprofen; +25 kV; 15 °C; hydrodynamic injection, 50 mbar for 3 s; UV detection at 230–265 nm.	Optimized Rs = 4.3 for fenoprofen, 4.8 for flurbiprofen, 4.4 for ibuprofen, 4.7 for indoprofen, 1.4 for ketoprofen, 8.2 for suprofen, and 5.8 for tiaprofenic acid; analysis time: 16.6–26.7 min.	[38]
CZE ILs + CDs	Carprofen, Ibuprofen, Indoprofen, Ketoprofen, Naproxen, Suprofen	Standard solutions	Uncoated or polybrene-coated fused-silica capillary, 35 cm × 50 μm i.d. (26.5 cm effective length); 5 or 60 mM acetate BGE, pH 5.0, in aqueous or H_2_O/MeOH (90:10 or 75:25, *v*/*v*) media, containing DM-β-CD or TM-β-CD and 10 mM EtChol NTf_2_ or PhChol NTf_2_; +25 kV; 25 °C; hydrodynamic injection, 30 mbar for 3 s; UV detection at 200–300 nm, depending on the analyte	Carprofen: Rs = 2.90 with TM-β-CD/EtChol NTf_2_ and 2.54 with TM-β-CD/PhChol NTf_2_ in H_2_O/MeOH (90:10, *v*/*v*). With the polybrene-coated capillary, maximum Rs values were 1.26 for carprofen, 0.88 for ibuprofen, 0.78 for indoprofen, 0.93 for ketoprofen, and 0.64 for suprofen; naproxen showed no enantioselectivity under any tested conditions.	[38]
NACE PA-β-CD	Flurbiprofen	Human plasma	Uncoated fused-silica capillary, 48.5 cm × 50 μm i.d. (40 cm effective length); 40 mM ammonium acetate BGE, pH 7.8, containing 10 mM PA-β-CD in MeOH; −25 kV; 15 °C; hydrodynamic injection, 50 mbar for 20 s; UV detection at 250 nm; flufenamic acid as internal standard; Oasis MAX SPE for sample preparation.	Rs = 4.8; linear range: 0.2–40 μg/mL; r^2^ = 0.9973 (S) and 0.9982 (R); LOD/LOQ: 0.06/0.20 μg/mL for both enantiomers; RSD: 1.1–4.1%; relative bias: −6.9 to 3.5% (S) and −5.1 to 1.4% (R); SPE recovery: 99% (*S*-flurbiprofen) and 98% (*R*-flurbiprofen).	[41]
CZE eremomycin	Fenoprofen, Flurbiprofen, Ibuprofen, Indoprofen, Ketoprofen	Standard solutions	Uncoated fused-silica capillary, 58.5 cm × 75 μm i.d. (50 cm effective length); 50 mM phosphate BGE, pH 6.5, containing 2.5 mM eremomycin; +10 kV; 20 °C; pressure injection, 12.5 kPa·s; external pressure, 1 kPa; UV detection at 230 nm.	Rs = 2.81 for fenoprofen, 7.11 for flurbiprofen, 3.91 for ibuprofen, 6.40 for indoprofen, and 5.50 for ketoprofen; α = 1.10–1.35.	[42]
CZE eremomycin	Fenoprofen, Flurbiprofen, Ibuprofen, Indoprofen, Ketoprofen	Standard solutions	Coupled chitosan-coated fused-silica capillary, 35 cm × 75 μm i.d. (27 cm effective length); 20 mM acetate BGE containing 5 × 10^−3^% chitosan and eremomycin: pH 4.5 with 0.75 mM eremomycin for flurbiprofen and indoprofen, pH 5.0 with 1.6 mM eremomycin for ketoprofen, and pH 6.2 with 1.6 mM eremomycin for fenoprofen and ibuprofen; −10 kV; 20 °C; pressure injection, 12.5 kPa·s; UV detection at 230 nm	Rs = 1.2 for fenoprofen, 2.6 for flurbiprofen, 5.3 for ibuprofen, 6.0 for indoprofen, and 4.1 for ketoprofen; α = 1.05–1.20; migration time <7 min	[43]
CZE eremomycin	Flurbiprofen, ketoprofen, fenoprofen, indoprofen	Standard solutions Ketoprofen gel	Uncoated fused-silica capillary, 45 cm × 50 μm i.d. (35.5 cm effective length); MeOH/50 mM phosphate BGE (60:40, *v*/*v*), pH 5.8, containing 2.0 mM eremomycin; −20 kV; hydrodynamic injection, 25 mbar for 15 s; UV detection at 254 nm	Rs = 2.01 for fenoprofen, 5.92 for flurbiprofen, 4.65 for indoprofen, and 3.80 for ketoprofen; analysis time <15 min. The water–MeOH BGE reduced eremomycin adsorption and Joule heating and improved migration-time and peak-area repeatability and baseline stability	[44]
EKC lysine-bridged hemispherodextrin (THLYSH)	Flurbiprofen, Ibuprofen, Suprofen, Tiaprofenic acid	Standard solutions	Uncoated fused-silica capillary, 61 cm × 75 μm i.d. (51 cm effective length); 33 mM sodium acetate–boric acid BGE, pH 6.2, containing 1 mM THLYSH; +25 kV; 20 °C; electrokinetic injection, 10 kV for 12 s; DAD detection.	Rs = 1.69 for flurbiprofen, 0.32 for ibuprofen, 0.45 for suprofen, and 4.23 for tiaprofenic acid.	[46]
OT-CEC using a graphene oxide-immobilized tentacle-type polymer stationary phase Me-β-CD	Naproxen, Pranoprofen, Warfarin	Standard solutions	Polymer-coated fused-silica capillary, 33 cm × 50 μm i.d. (24.5 cm effective length); 30 mM sodium citrate–citric acid BGE, pH 5.0, containing 20 mM Me-β-CD for naproxen and pranoprofen, with 20% ACN for pranoprofen; +20 kV; 20 °C; hydrodynamic injection, 50 mbar for 3 s; UV detection at 235 nm for naproxen and 210 nm for pranoprofen.	Rs = 1.56 for naproxen (α = 1.047) and 1.51 for pranoprofen (α = 1.065); RSD < 2.4% for migration time and <3.7% for Rs.	[47]
CE-UV Vancomycin	Ibuprofen	Pharmaceutical tablets; urban water; human urine	Uncoated fused-silica capillary, 60 cm × 50 μm i.d. (47 cm effective length); 100 mM phosphate BGE, pH 6.5, containing 1 mM vancomycin; +20 kV; 20 °C; hydrodynamic injection, 65 mbar for 10 s; UV detection at 214 nm.	Linear range: 0.125–5.0 μg/mL (R^2^ = 0.999); LOD/LOQ: 0.025/0.070 μg/mL; precision: RSD < 2.3%; recovery: 93.2%; preconcentration factor: 33.4.	[49]
CEC imidazole/β-CD covalently coated capillary	Ketoprofen	Standard solutions	Covalently coated fused-silica capillary, 60 cm × 50 μm i.d. (50 cm effective length), with an imidazole/β-CD coating prepared using 750 mg/mL Ts-β-CD; 20 mM NaH_2_PO_4_ BGE, pH 2.0; −20 kV; 20 °C; hydrodynamic injection, 30 mbar for 5 s; UV detection at 254 nm	Baseline enantioseparation of ketoprofen; α = 1.03.	[50]
CZE-UV Tri-OMe-β-CD	Ibuprofen	Dexibuprofen; mechanically stressed dexibuprofen samples	Uncoated fused-silica capillary, 60.2 cm × 75 μm i.d. (50.0 cm effective length); 50 mM sodium acetate BGE, pH 5.0, containing 10 mM magnesium acetate and 35 mM Tri-OMe-β-CD; +26 kV; 27 °C; hydrodynamic injection, 3.45 kPa for 5 s; DAD detection at 202 nm.	Rs ≥ 2.3; linear range: 0.68–5.49% *R*-ibuprofen (R^2^ = 0.9991); LOQ: 0.21% *R*-ibuprofen (1.25 μg/mL) and 0.15% using racemic ibuprofen dilution; recovery: 97–103%; repeatability/intermediate precision RSD: 1.52/1.97%.	[51]
CZE-UV Vancomycin HP-β-CD	Naproxen	Standard solutions	Uncoated fused-silica capillary, 60 cm × 50 μm i.d. (47 cm effective length); vancomycin system: phosphate BGE, pH 6.0, containing 0.5 mM vancomycin, +22 kV, 15 °C; HP-β-CD system: phosphate BGE, pH 5.0, containing 7.71 mg/mL HP-β-CD, +14 kV, 15 °C; hydrodynamic injection, 65 mbar for 10 s; UV detection at 230 nm	Baseline enantioseparation with both CSs; linear range: 1–50 μg/mL; LOQ: 1.0 μg/mL with vancomycin and 0.5 μg/mL with HP-β-CD; precision: RSD 8.6% and 15.1%, respectively.	[52]
CEC HP-β-CD	Carprofen, Flurbiprofen, Ibuprofen, Ketoprofen, Loxoprofen	Standard solutions	BSA/pepsin mixed monolithic CSP; 10 mM phosphate BGE, pH 7.0, containing 50 mM HP-β-CD; +20 kV; room temperature; UV detection at 230 nm.	Rs = 5.09 for carprofen, 6.01 for flurbiprofen, 4.48 for ibuprofen, 5.65 for ketoprofen, and 4.26 for loxoprofen; run-to-run, intra-day, and inter-day RSD < 5%; column-to-column RSD < 6%.	[53]
OT-CEC {(HQA)(ZnCl_2_)(2.5H_2_n__O)} chiral MOF stationary phase	Ibuprofen	Standard solutions	{(HQA)(ZnCl_2_)(2.5H_2_n__O)}-coated fused-silica capillary, 50 cm × 75 μm i.d. (41.5 cm effective length); 20 mM phosphate BGE, pH 6.75, containing 10% MeOH; +15 kV; 25 °C; UV detection at 254 nm.	Rs = 2.71; t_1_ = 15.45 min; t_2_ = 16.73 min; no enantioseparation with the bare capillary; run-to-run RSD < 1.74%, day-to-day RSD < 1.66%, column-to-column RSD < 3.40% for migration time.	[54]
CE BSA-AuNP as QSP	Fenoprofen, Flurbiprofen, Ibuprofen, Indoprofen, Ketoprofen, Pyranoprofen	Standard solutions	15 mM phosphate BGE, pH 8.0, containing 20 mM BSA and 3.2 nM AuNPs; +16 kV; 25 °C; UV detection at 210 nm.	Rs = 15.21 for fenoprofen, 9.83 for flurbiprofen, 8.15 for ibuprofen, 13.83 for indoprofen, 10.02 for ketoprofen, and 5.34 for pyranoprofen; intra-day, inter-day, and inter-column RSD ≤ 5.9%.	[55]
CE physically adsorbed chitosan-coated capillary Vancomycin	Ketoprofen	Standard solutions	Chitosan-coated fused-silica capillary, 60 cm × 50 μm i.d. (50 cm effective length); 25 mM phosphate BGE, pH 4.2, containing 2.5 mM vancomycin and 10% MeOH; −20 kV; 20 °C; UV detection at 230 nm.	Ketoprofen: Rs = 3.0 with the chitosan-coated capillary vs. 1.4 with the uncoated capillary under the same BGE conditions.	[56]

Abbreviations: ACN, acetonitrile; AuNPs, gold nanoparticles; BGE, background electrolyte; BSA, bovine serum albumin; CD, cyclodextrin; β-CD, β-cyclodextrin; CE, capillary electrophoresis; CEC, capillary electrochromatography; CGE, capillary gel electrophoresis; CM-β-CD, carboxymethyl-β-cyclodextrin; CS, chiral selector; CSP, chiral stationary phase; CZE, capillary zone electrophoresis; DAD, diode-array detection; Di-OMe-β-CD, heptakis(2,6-di-O-methyl)-β-cyclodextrin; DMEB, (1R,2S)-N-dodecyl-N-methylephedrinium bromide; DSPE, dispersive solid-phase extraction; EtChol NTf_2_, ethylcholine bis(trifluoromethylsulfonyl)imide; GO, graphene oxide; HDB, hexadimethrine bromide; Hepta-MeNH-β-CD, heptamethylamino-β-cyclodextrin; HP-β-CD, hydroxypropyl-β-cyclodextrin; HP-γ-CD, hydroxypropyl-γ-cyclodextrin; HS-β-CD, heptakis-6-sulfato-β-cyclodextrin; IL, ionic liquid; IPA-β-CD, 6-monodeoxy-6-mono(2-hydroxy)propylamino-β-cyclodextrin; LOD, limit of detection; LOQ, limit of quantification; Me-β-CD, methyl-β-cyclodextrin; MeOH, methanol; MEKC, micellar electrokinetic chromatography; MES, 2-(N-morpholino)ethanesulfonic acid; NACE, nonaqueous capillary electrophoresis; OT-CEC, open-tubular capillary electrochromatography; PA-β-CD, 6-monodeoxy-6-mono(3-hydroxy)propylamino-β-cyclodextrin; PhChol NTf_2_, phenylcholine bis(trifluoromethylsulfonyl)imide; PMMA-β-CD, permethyl-6-monoamino-6-monodeoxy-β-cyclodextrin; PVA, poly(vinyl alcohol); QA-β-CD, quaternary ammonium β-cyclodextrin; QSP, quasi-stationary phase; RSD, relative standard deviation; SBE-β-CD, sulfobutyl ether-β-cyclodextrin; SPE, solid-phase extraction; TBAH, tetrabutylammonium hydroxide; TM-β-CD, trimethyl-β-cyclodextrin; Tri-OMe-β-CD, heptakis(2,3,6-tri-O-methyl)-β-cyclodextrin; Ts-β-CD, tosyl-β-cyclodextrin; UV, ultraviolet.

## Data Availability

No new data were created or analyzed in this study. Data sharing is not applicable to this article.

## Abbreviations

The following abbreviations are used in this manuscript: ACN, acetonitrile; AuNPs, gold nanoparticles; BGE, background electrolyte; BSA, bovine serum albumin; CCD, central composite design; CD, cyclodextrin; β-CD, β-cyclodextrin; CE, capillary electrophoresis; CE-CD, capillary electrophoresis–circular dichroism; CEC, capillary electrochromatography; CGE, capillary gel electrophoresis; CM-β-CD, carboxymethyl-β-cyclodextrin; COX, cyclooxygenase; CS, chiral selector; CSP, chiral stationary phase; CZE, capillary zone electrophoresis; DAD, diode-array detection; Di-OMe-β-CD, heptakis(2,6-di-O-methyl)-β-cyclodextrin; DM-β-CD, dimethyl-β-cyclodextrin; DMEB, (1*R*,2*S*)-N-dodecyl-N-methylephedrinium bromide; DoE, design of experiments; DSPE, dispersive solid-phase extraction; EA-β-CD, 6-monodeoxy-6-mono(2-hydroxy)ethylamino-β-cyclodextrin; EOF, electroosmotic flow; EtChol NTf_2_, ethylcholine bis(trifluoromethylsulfonyl)imide; GO, graphene oxide; HDB, hexadimethrine bromide; Hepta-MeNH-β-CD, heptamethylamino-β-cyclodextrin; HP-β-CD, hydroxypropyl-β-cyclodextrin; HP-γ-CD, hydroxypropyl-γ-cyclodextrin; HS-β-CD, heptakis-6-sulfato-β-cyclodextrin; IL, ionic liquid; IPA-β-CD, 6-monodeoxy-6-mono(2-hydroxy)propylamino-β-cyclodextrin; LLE, liquid–liquid extraction; LOD, limit of detection; LOQ, limit of quantification; Me-β-CD, methyl-β-cyclodextrin; MEKC, micellar electrokinetic chromatography; MeOH, methanol; MES, 2-(N-morpholino)ethanesulfonic acid; MOF, metal–organic framework; NACE, nonaqueous capillary electrophoresis; NSAIDs, nonsteroidal anti-inflammatory drugs; OT-CEC, open-tubular capillary electrochromatography; PA-β-CD, 6-monodeoxy-6-mono(3-hydroxy)propylamino-β-cyclodextrin; PhChol NTf_2_, phenylcholine bis(trifluoromethylsulfonyl)imide; PMMA-β-CD, permethyl-6-monoamino-6-monodeoxy-β-cyclodextrin; PVA, poly(vinyl alcohol); QA-β-CD, quaternary ammonium β-cyclodextrin; QSER, quantitative structure–enantioselectivity relationships; QSP, quasi-stationary phase; R, resolution; RSD, relative standard deviation; SBE-β-CD, sulfobutyl ether-β-cyclodextrin; SPE, solid-phase extraction; TBAH, tetrabutylammonium hydroxide; TDDFT, time-dependent density functional theory; THLYSH, lysine-bridged hemispherodextrin; TM-β-CD, trimethyl-β-cyclodextrin; Tri-OMe-β-CD, heptakis(2,3,6-tri-O-methyl)-β-cyclodextrin; Ts-β-CD, tosyl-β-cyclodextrin; UV, ultraviolet.
